# In vivo study of hepatic oxidative stress and mitochondrial function in rabbits with severe hypotension after propofol prolonged infusion

**DOI:** 10.1186/s40064-016-2970-2

**Published:** 2016-08-15

**Authors:** Sónia Campos, Luís Félix, Carlos Venâncio, Maria de Lurdes Pinto, Francisco Peixoto, Paula Guedes de Pinho, Luís Antunes

**Affiliations:** 1 Centre for the Research and Technology of Agro-Environmental and Biological Sciences (CITAB) and Veterinary Sciences Department, University of Trás-os-Montes and Alto Douro (UTAD), Quinta de Prados, Apartado 1013, 5001-801 Vila Real, Portugal; 2 Institute for Research and Innovation in Health (i3S), Laboratory Animal Science, Institute of Molecular and Cell Biology (IBMC), University of Porto, Rua Alfredo Allen, 208, 4200-135 Porto, Portugal; 3 UCIBIO@REQUIMTE-Laboratory of Toxicology, Department of Biological Sciences, Faculty of Pharmacy, University of Porto, Porto, Portugal; 4 Life Sciences and Environment School (ECVA), Department of Chemistry, University of Trás-os-Montes e Alto Douro, Vila Real, Portugal

**Keywords:** Propofol, Rabbit anesthesia, Mitochondrial bioenergetics, Propofol infusion syndrome (PRIS)

## Abstract

In humans, prolonged sedations with propofol or using high doses have been associated with propofol infusion syndrome. The main objective of this study was to evaluate the effects of prolonged high-dose administration of a specific propofol emulsion (Propofol Lipuro) and an improved lipid formulation (SMOFlipid) in liver mitochondrial bioenergetics and oxidative stress of rabbits, comparatively to a saline control. Twenty-one male New Zealand white rabbits were randomly allocated in three groups that were continuously treated for 20 h. Each group of seven animals received separately: NaCl 0.9 % (saline), SMOFlipid (lipid-based emulsion without propofol) and Lipuro 2 % (propofol lipid emulsion). An intravenous propofol bolus of 20 mg kg^−1^ was given to the propofol Lipuro group to allow blind orotracheal intubation and mechanical ventilation. Anesthesia was maintained using infusion rates of: 20, 30, 40, 50 and 60 mg kg^−1^ h^−1^, according to the clinical scale of anesthetic depth and the index of consciousness values. The SMOFlipid and saline groups received the same infusion rate as the propofol Lipuro group, which were infused during 20 consecutive hours. At the end, the animals were euthanized, livers collected and mitochondria isolated by standard differential centrifugation. Mitochondrial respiration, membrane potential, swelling and oxidative stress were evaluated. Data were processed using one-way ANOVA (p < 0.05). The animals revealed a significant decrease in cardiovascular parameters showing bradycardia and severe hypotension. No statistical differences were observed when using pyruvate as substrate, however, when using succinate as respiratory substrate, significant decrease in ADP-stimulated respiration rate was observed for SMOFlipid group (p = 0.002). Lipid peroxides (p < 0.01) and protein carbonyls (p = 0.01) showed a statistically significant difference between propofol Lipuro and the SMOFlipid groups. These results suggest that lipid-based emulsions can be involved in the regulation of different pathways that ultimately lead to a decrease of state 3 mitochondrial respiration rate. The infusion of propofol Lipuro during prolonged periods, in addition to marked hypotension and hypoperfusion, also showed to have higher anti-oxidant activity and lower impairment of the mitochondrial function comparatively to the improved lipid formulation, SMOFlipid, using the rabbit as animal model.

**Electronic supplementary material:**

The online version of this article (doi:10.1186/s40064-016-2970-2) contains supplementary material, which is available to authorized users.

## Background

Propofol is a hypnotic drug largely used through the last decades for both human and animal anesthesia and sedation of healthy and critical patients (Li et al. 2012). This small lipophilic molecule holds favorable pharmacokinetic and pharmacodynamic properties as rapid onset, short clinical effect and fast recovery that turn it the choice intravenous (IV) drug for continuous infusion for the most diverse procedures (Fodale and La Monaca 2008). Nonetheless, adverse side effects were observed when propofol was administered in high doses or for long uninterrupted periods to humans, especially, children (>4 mg kg^−1^ h^−1^; >48 h) (Kam and Cardone 2007; Ahlen et al. 2006; Agrawal et al. 2013) and rabbits (>20 mg kg^−1^ h^−1^; >20 h) (Ypsilantis et al. 2007), indicating the presence of a complex set of signs and symptoms that owned the name of propofol infusion syndrome (PRIS). The clinical features of PRIS include bradycardia and cardiac asystole, hypotension, lipemic plasma, fatty liver, metabolic acidosis and renal failure that can lead to death (Kam and Cardone 2007; Vasile et al. 2003; Campos 2010). The mechanisms and the physiopathology of this syndrome have not been completely elucidated but in vitro and in vivo studies suggested dysfunction of the mitochondrial respiratory chain, impaired fatty acid oxidation and unbalancing energy production (Kam and Cardone 2007; Branca et al. 1991a; Schenkman and Yan 2000; Wolf et al. 2001). Decreased generation and/or maintenance of the transmembrane electrical potential as well as reduced cytochrome C oxidase activity have also been reported in the presence of propofol (Branca et al. 1991b). Genetic susceptibility to propofol, such as an inherited error of the mitochondrial fatty acid oxidation and polymorphisms in the enzymes involved in the propofol metabolism may also play a role (Zaccheo and Bucher 2008). The real contribution of propofol to PRIS has been discussed, as it can result from other causes (inherent diseases as liver failure or genetic polymorphisms) that also conduct to the manifestation of fatal clinical signs that occur while administering propofol or other concomitant drugs (Fudickar and Bein 2009). Propofol-sedated rabbits evidenced multi-organ dysfunction syndrome similar to human PRIS and, recently, propofol-sedated rats indicated a possible involvement of coenzyme Q in the physiopathology of PRIS (Ypsilantis et al. 2007; Vanlander et al. 2014). The present study aims to evaluate the in vivo effects on hepatic mitochondrial bioenergetics and oxidative stress parameters after a long-term high-dose administration of a specific propofol formulation (Propofol Lipuro) and an improved lipid emulsion (SMOFlipid) on rabbits.

## Methods

### Animals

All procedures related to animals were carried out under university ethics committee authorization and project license approved by the national regulatory office: Direcção Geral de Alimentação e Veterinária (DGAV), protocol No 0420/000/000/2012. All people involved in animal procedures had appropriate training following the FELASA recommendations and were accredited by DGAV. This study was conducted in agreement with the national animal welfare laws, guidelines and policies for care and use of experimental animals.

Twenty-one male New Zealand white rabbits, in-house bred and average weight 2.74 ± 0.21 kg were used. Animals were daily handled from 3 weeks old by the researcher who performed the anesthetic procedure for habituation till approximately 3 months old, when the experiment was carried out. They were also contained (2 h per day) in a standard box during 2 weeks prior to the experiment. Animals were housed in proper standard cages, in a room with controlled temperature (20–22 °C), humidity (50–60 %), ventilation and light/dark (12/12 h) cycle. Commercial pellet food and water were provided ad libitum, with exception to the last 4 h prior to the study. Animal welfare, behavior and clinical signs were periodically assessed before the experimental study beginning.

### Anesthesia and monitoring

Animals were randomly allocated into three groups, containing seven rabbits each: the propofol Lipuro (Propofol Lipuro® 2 %, B. Braun, Germany), the SMOFlipid emulsion (SMOFlipid® 20 %, Fresenius Kabi Pharma, Sweden) and the saline (NaCl 0.9 %, B. Braun, Germany) group. Monitoring of anesthesia was ensured by Datex S/5 Anesthetic station (Datex Ohmeda, Helsinki, Finland) and included cardiorespiratory parameters: heart rate (HR), mean arterial blood pressure (MABP), systolic arterial blood pressure (SABP), diastolic arterial blood pressure (DABP), end-tidal CO_2_ (ETCO_2_), arterial blood oxygen saturation (SpO_2_), respiratory rate (RR) and rectal temperature (T). The evaluation of the anesthetic depth was performed by assessing the main reflexes (palpebral, corneal and pedal withdrawal) (Silva et al. 2011) and by using the IoC-View monitor (Aircraft Medical, Barcelona, Spain) to ensure the right level of anesthesia in all animals. Only the animals of the propofol group were under anesthesia, the animals of the SMOFlipid and saline control stayed awake during the entire experiment in proper restraining cages so they could adequately receive the intravenous infusion.

After baseline recording in the awake animals during 5 min, the fur on the ears was clipped and a local analgesic cream was applied to the ears skin (EMLA, Nycomed US Inc, New York, USA). Twenty minutes later, 22G catheters were placed in the marginal ear veins (for propofol delivery and blood samples collection) and in the central ear arteries (for invasive blood pressure monitoring and samples for blood gases measurements). In the propofol group, the animals were pre-oxygenated with a facial mask at 5 L min^−1^ for 5 min. Anesthesia was induced with a 20 mg kg^−1^ bolus dose of propofol Lipuro, using a syringe pump (Asena GH, Alaris Medical Systems, USA) at an infusion rate (IR) of 200 ml h^−1^. Following blind orotracheal intubation with a cuffed endotracheal tube with 2.5 mm internal diameter for mechanical ventilation and 100 % oxygen support, the rabbits were positioned in right recumbence above a heating blanket. Mechanical ventilation was performed by pressure (8–10 cmH_2_O) using Datex S/5 Anesthetic station (*Datex* Ohmeda, Helsinki, Finland). The initial settings (respiratory rate 20–25 breath min^−1^; peak inspiratory pressure 10–12 cmH_2_O; oxygen flow rate 1–3 L min^−1^; positive end-expiratory pressure of 3–5 cmH_2_O; 1 inspiration: 2 expirations) ensured a tidal volume of 6–8 mL/kg, and these parameters were adjusted to maintain a PaCO_2_ between 35 and 45 mmHg, if required. A deep inspiration (30 cmH_2_O for 1 s) was performed every 2 h, in addition to the applied positive end-expiratory pressure (PEEP) to avoid atelectasis. Recumbence was also changed every 4 h. Propofol Lipuro administration started with an infusion rate of 60 mg kg^−1^ h^−1^ as tolerance to propofol was described in the first 2 h after the onset of the infusion and also due to propofol rapid metabolization and high clearance observed during enzymatic activation (Campos and al 2015). In each anesthetized animal, the level of anesthesia was assured by evaluation of the main reflexes (Silva et al. 2011) and according to the IoC values which were maintained between the 50 and 70. Reflexes evaluation was always performed at specific time points of the study by the same investigator who was blinded to procedures. All infusion rates varied from 20 to 60 mg kg^−1^ h^−1^, changed in steps of 10 mg kg^−1^ h^−1^, every time the animals from the propofol Lipuro group showed signs of awakening or deepening from anesthesia, so a similar level of anesthetic depth could be maintained over time. These doses were defined in agreement with previous data collected from rabbits sedated only with propofol, without pre-medication and for organ toxicity and electroencephalographic purposes (Ypsilantis et al. 2007; Silva et al. 2011; Campos et al 2015; Martin-Cancho et al. 2006). Adjustments in the infusion rate were always performed according to clinical reflexes, IoC values and cardiorespiratory signs. Whenever animals showed higher or lighter sedation, the infusion rate was readjusted to desired level. Infusion rates started at the same time in all groups, with the SMOFlipid and saline groups always receiving the same infusion rate as the propofol Lipuro group. Anesthesia was maintained during 20 consecutive hours and vital signs evaluation was performed continuously but only recorded every 3 h (T0, T3, T6, T9, T12, T15, T18) and at the end (T20), before blood samples collection. At the end of the experiment, the animals from the propofol Lipuro group were euthanized by exsanguination under anesthesia and the animals from the SMOFlipid and saline groups were sacrificed by cerebral concussion followed by exsanguination. Livers from all animals were immediately collected for mitochondria isolation.

### Blood and organ samples

Blood samples (2 mL per time-point) were collected in all animals before the infusion protocol (baseline data) and every 3 h till the end (T20) for biochemical analysis (serum was separated by centrifugation at 3000 rpm for 15 min and stored at −20 °C till analysis), for blood gases measurement and for propofol quantification (arterial blood samples into heparinized syringes and tubes, respectively). Blood biochemical analysis were performed using an ABX Pentra 400 apparatus (Horiba ABX SAS, Montpellier, France) and included: hematocrit, total proteins (TP), urea, creatinine, aspartate aminotransferase (AST), alanine aminotransferase (ALT), lactate dehydrogenase (LDH), total bilirubin (TB), direct bilirubin (DB), alkaline phosphatase (ALP), cholesterol, triglycerides, amylase, creatine kinase (CK), glucose, potassium (K^+^), sodium (Na^+^), total calcium (Ca^2+^), magnesium (Mg^2+^), phosphorus and chloride (Cl^−^).

Arterial blood gases analysis included: pH, PaCO_2_, PaO_2_, ${\text{HCO}}_{3}^{ - }$ and oxygen saturation. A blood gas/electrolyte/co-oximetry analyzer (GEM Premier 3000, Bruxelles, Belgium) was used.

Liver, heart, kidney, lungs, skeletal muscle and urinary bladder samples were taken from each animal and fixed in 10 % neutral buffered formol-saline solution by immersion and processed for histopathology using routine techniques for paraffin embedding and sliced at 3 µm pieces and primarily stained with hematoxylin-eosin (H&E) for histopathological analysis. At the same time, liver was preserved at −80 °C for propofol quantification (Campos and al 2014).

#### Propofol quantification in plasma and liver

Propofol concentration at T20 was obtained by gas chromatography/ion trap-mass spectrometry (GC/IT-MS) in plasma and liver samples using a GC Varian CP-3800 (California, USA) equipped with a selective ion trap mass detector (Varian Saturn 4000, California USA) using methods previously validated by our group (Campos and al 2014).

#### Mitochondria isolation

Mitochondria were isolated from rabbit liver by standard differential centrifugation, in agreement with standardized methods (Gazzotti et al. 1997) with few adjustments. Briefly, the whole liver was rapidly withdrawn, washed, and homogenized in ice-cold homogenization medium [250 mM sucrose, 5 mM HEPES, 0.1 mM EDTA, and 0.1 % defatted bovine serum albumin (BSA), pH 7.4]. Tissue and nuclei were separated by centrifugation at 1000×*g* for 10 min at 4 °C (Sigma 3K-16). Mitochondria were recovered from the supernatant by centrifugation at 10,000×*g* for 10 min at 4 °C. The brown mitochondrial pellet was washed twice, re-suspended in respiratory buffer (70 mM sucrose, 220 mM mannitol, 2 mM HEPES, 0.5 mM EDTA, pH 7.4) and divided in two aliquots, one immediately used and the other frozen at −80 °C for further analysis. Total protein content of samples was determined by Biuret method (Gornall et al. 1949) using BSA as standard.

### Mitochondria respiration

Oxygen consumption of isolated liver mitochondria was monitored polarographically (Estabrook 1967) with a Clark-type oxygen electrode at 25 °C, in a 1 mL water thermostatic incubation stirred chamber (CB1-D Hansatech), with constant stirring. The standard reaction medium consisted of 130 mM sucrose, 50 mM KCl, 5 mM MgCl_2_, 5 mM KH_2_PO_4_ and 5 mM HEPES at pH 7.2 on the basis of 1 mg mitochondrial protein per 1 mL buffer. After a 2 min equilibration period, mitochondria were energized with pyruvate–malate (5 mM) or succinate (5 mM) as substrates. Pyruvate–malate indirectly supplies electrons to mitochondrial complex I and succinate directly reduces complex II. Rotenone (3 µM) was used to inhibit complex I and avoid reverse flow of electrons from complex II to complex I. State 4 respiration corresponds to resting oxygen consumption after phosphorylation of a small amount of ADP. State 3 was started by adding ADP (100 nmol mg protein^−1^). Uncoupled respiration was initiated by the addition of carbonylcyanide *p*-trifluoromethoxyphenylhydrazone (FCCP) (1 µg mL^−1^). The rate of oxygen uptake by mitochondria is expressed as nmol O_2_ min^−1^ mg protein^−1^. Respiratory control ratio (RCR) and ADP/O ratios were calculated (Chance and Williams 1956) in mitochondria energized with pyruvate–malate and succinate.

#### Mitochondrial Membrane Potential (ΔΨ)

Mitochondrial transmembrane potential (ΔΨ) was measured by a flourimetric method using safranin O dye (Akerman and Wikstrom 1976) and recorded in a Varian Cary Eclipse fluorescence spectrophotometer (Varian Inc, Palo Alto, CA, USA) operating at 485 nm excitation and 586 nm emission at 25 °C. Accumulation of safranine O dye in mitochondria is driven by membrane potential and results subsequently in decrease of fluorescence. Depolarization results in the release of safranine O dye from mitochondria and subsequent increase of fluorescence. Freshly isolated mitochondria (0.5 mg protein) were suspended in 3 mL of a medium (pH 7.4) containing 75 mM sacarose, 50 mM KCl, 30 mM Tris, 2 mM KH_2_PO_4_, 2 mM MgCl_2_ and 10 μM EGTA in the presence of 5 μM safranine O dye supplemented with 1 μg mL^−1^ rotenone. The formation of membrane potential was induced after 2 min equilibration by the addition of 10 mM succinate as substrate. Data obtained were calibrated using a K^+^ gradient (Akerman and Wikstrom 1976). To this end, safranine O fluorescence was recorded in the presence of 2 nM valinomycin and stepwise increasing K^+^ concentrations (0.2–120 mM) which allowed calculation of ΔΨ by the Nernst equation assuming a matrix K^+^ = 150 mm (Akerman and Wikstrom 1976). No correction for bound safranine O dye was applied because titration of the fluorescence signal with the dye in buffer without or with sample resulted in undistinguishable calibration curves.

### Mitochondrial swelling

Swelling of isolated mitochondria was measured by absorbance changes at 540 nm on a Varian 50 spectrophotometer (Varian Inc, Palo Alto, CA, USA), in a thermostatic chamber with magnetic stirring at 30 °C according to Oliveira et al. (2001). Briefly, 0.5 mg mitochondrial protein was added to 2 mL of medium containing 135 mM potassium acetate, 5 mM HEPES (pH 7.2), 0.1 mM EGTA, and 0.2 mM EDTA supplemented with 2 μM rotenone. All assays were performed in the presence of 1 μg/mL valinomycin to increase permeability to potassium. A control assay was performed in the presence of 1 μM FCCP as a calibration standard to cause maximal swelling.

### Mitochondrial respiratory chain complex activities

Aliquots of mitochondrial suspensions were submitted to three cycles of freezing–thawing in order to disrupt intact mitochondria and measures were started by the addition of 10 μg of mitochondrial protein on a temperature controlled (30 °C) Biotek Power Wave XS2 well plate reader (Bio-Tek Instruments, Winooski, VT, USA) according to previously established methods (Kiebish et al. 2008). Complex I (NADH: ubiquinone oxidoreductase) activity was monitored following the oxidation of NADH at 340 nm (ε = 6220 M^−1^ cm^−1^) in a medium containing 50 mM potassium phosphate, pH 7.4, 2 mM KCN, 5 mM MgCl_2_, 2.5 mg mL^−1^ BSA, 0.03 mM antimycin, 0.1 mM decylubiquinone and 0.3 mM NADH. Only the rotenone sensitive activity was considered. Complex II (succinate dehydrogenase) activity was monitored at 600 nm by following the reduction of 6,6-dichlorophenolindophenol (DCPIP) (ε = 19,100 M^−1^ cm^−1^) in a buffer containing 50 mM potassium phosphate, pH 7.4, 20 mM succinate, 2 mM KCN, 50 μM DCPIP, 0.025 mM rotenone, and 0.03 mM antimycin. Only the oxaloacetate sensitive rate was considered. Complex III (cytochrome *bc*_1_ complex) activity was monitored at 550 nm by following the ubiquinol reduction of cytochrome *c* (ε = 18,500 M^−1^ cm^−1^) in a buffer containing 5 mM potassium phosphate, pH 7.4, 1 mM EDTA, 1 mM KCN, 0.1 % tryton X-100, 32 μM oxidized cytochrome *c* and 35 μM decylubiquinol, prepared according to Luo et al. (2008). Only the antimycin sensitive rate was considered. Complex IV (cytochrome *c* oxidase) activity was determined at 550 nm by measuring the oxidation of reduced ferrocytochrome *c* (ε = 18,500 M^−1^ cm^−1^), prepared according to Trounce et al. (1996), in a buffer containing 10 mM Tris–HCl and 120 mM KCl, pH 7.0. Only the cyanide sensitive rate was considered. Activities were normalized to citrate synthase activity which was performed at 412 nm following the reduction of 5,5′-dithio-bis(2-nitrobenzoic acid) (ε = 13,600 M^−1^ cm^−1^) in a buffer containing 200 mM Tris–HCl, pH 8.0, 0.01 mM DTNB, 0.02 % Triton X-100, 1 mM oxaloacetate and 0.37 mM Acetil-CoA (Monteiro-Cardoso et al. 2015).

### Hepatic oxidative stress assay

The enzymatic and non-enzymatic antioxidant activities were measured at 25 °C in the hepatic cytosolic fraction from all groups considered in this study. Livers were homogenized in ice-cold potassium phosphate buffer 50 mM, pH 7.0. The fractions were obtained after centrifugation at 16,000×*g* for 20 min at 4 °C (Sigma 3K-16). Superoxide dismutase (SOD) activity was assayed by measuring its ability to inhibit the photochemical reduction of nitrobluetetrazolium (NBT) at 560 nm (Paya et al. 1992), in the presence of 100 mM potassium phosphate buffer (pH 7.8), 10 mM EDTA, 10 mM NBT, 10 mM hypoxanthine and 0.023 U mol^−1^ xanthine oxidase. One unit of SOD was defined as the enzyme activity that inhibited the photo-reduction of NBT to blue formazan by 50 %. Catalase (CAT) activity was evaluated following oxygen production in the system using a Clark’s oxygen electrode (Del Rio et al. 1977) in a 50 mM sodium phosphate buffer (pH 7.0) containing 1 M H_2_O_2_. CAT activity was expressed in terms of micromoles H_2_O_2_ consumed per minute per milligram protein. Both GSH and GSSG, glutathione in reduced and oxidized states respectively, were measured fluorometrically using the fluorochrome ortho-phthalaldehyde (OPA) (1 mg mL^−1^ methanol) at 350 and 420 nm excitation and emission wavelengths, respectively (Hissin and Hilf 1976). To measure GSH content, samples were incubated at room temperature in a buffer containing 100 mM potassium phosphate buffer (pH 8.0), 5 mM EDTA and 20 μL OPA. To determinate GSSG content, samples were first incubated at room temperature with 0.04 M of *N*-ethylmaleimide (NEM) for 30 min and then incubated in a medium containing 0.1 N NaOH (pH 12.0) and 20 μL OPA. Concentrations were calculated using standard curves prepared with different concentrations of GSH and GSSG and expressed as their ratio. The lipid peroxidation (LPO) was evaluated in terms of thiobarbituric acid reactive substance (TBARS) formation (Ottolenghi 1959). The data is expressed as nmol MDA formed per milligram protein using molar extinction coefficient of 1.56  ×  10 M^−1^ cm^−1^. The estimation of lipid hydroperoxides was assayed by the method of Jiang et al. (1992). Protein carbonyls were measured using the method of Reznick and Packer (1994) by reaction with 10 mM 2,4-dinitrophenylhydrazine (DNPH). ROS generation was measured in liver samples according to a method already described in literature (Monteiro-Cardoso et al. 2015). Total protein content of samples was determined by Biuret method (Gornall et al. 1949) using BSA as standard.

### Statistical analysis

Animal sample size was calculated using a statistical power test with a power above 80 % and α = 0.05, where mean and standard deviation information for the different variables were obtained from previous works with rabbits (Campos 2010; Silva et al. 2011). Data was imported to GraphPad Prism® software (GraphPad Software, version 6.0, Inc., San Diego, CA, USA). A Shapiro–Wilk test was used to evaluate data normality. Clinical and mitochondria data that follow normal distribution are showed as mean ± standard deviation (M ± SD) and whereas results from non-parametric data are showed as median and interquartile range. To assess the variability from cardiorespiratory data and blood biochemical analysis, statistical comparisons were performed between the defined study times (T0–T20) using analysis of variance (ANOVA) for repeated measures with the Tukey’s correction for normal data and the Friedman test with Dunn’s post hoc for non-parametric data. Statistical significance between groups for mitochondria sample analysis was determined using one-way ANOVA followed by Tukey’s or the Kruskal–Wallis’ followed by Dunn’s multiple comparison tests, according to data normality. For all analyses, values of p < 0.05 were considered significant.

## Results

### Clinical parameters and propofol concentration

All anesthetized animals survived the 20 h of continuous anesthesia. The mean infusion rate of propofol was 37.6 ± 18.1 mg kg^−1^ h^−1^ and the first evidences of collapsing were observed at 12 ± 2.4 h after the infusion onset and included significant variations in cardiorespiratory signs (Table 1) and in blood biochemistry measurements in the propofol Lipuro group (Additional file 1: Table S1). Changes in cardiorespiratory parameters were essentially noticed after 12 h of propofol Lipuro administration. Heart rate and blood pressure were the variables that showed significant variations during the anesthetic period, particularly, after 12 h of infusion. Hypotension was marked in the end of the infusion protocol (T20) and was accompanied by an increase in heart rate, however, only slight drops in pH, HCO^−3^, T °C and SpO_2_ were noticed, with an increase in PaCO_2_ within the physiological limits. Clinical signs were also monitored in the saline and SMOFlipid groups and deviations from the normal ranges of each parameter were not observed.

**Table 1:** Clinical data from anesthetized animals (propofol Lipuro group) during the 20 h of anesthesia

Parameter	Time-point (h)							
	T0	T3	T6	T9	T12	T15	T18	T20
pH	7.41 ± 0.03	7.39 ± 0.05	7.36 ± 0.05	7.36 ± 0.07	7.34 ± 0.03	7.33 ± 0.05	7.29 ± 0.06	7.26 ± 0.02
PaCO_2_	35.6 ± 1.2	36.3 ± 2.1	37.0 ± 2.7	40.2 ± 0.9	39.7 ± 1.4	44.3 ± 1.7	45.1 ± 2.2	46.5 ± 1.8*
PaO_2_	98.4 ± 5.6	109.9 ± 10.4	118.3 ± 12.6	134.1 ± 15.6	129.8 ± 22.8	111.4 ± 19.2	102.8 ± 13.6	96.4 ± 9.6
HCO_3_^−^	20.6 ± 1.1	21.9 ± 2.3	21.2 ± 3.1	20.8 ± 3.3	19.9 ± 1.7	19.3 ± 2.4	18.6 ± 3.1	17.1 ± 1.6
HR	243.0 ± 18.2	210.6 ± 11.6	196.2 ± 10.2*	191.6 ± 19.5*	191.1 ± 15.2*	198.9 ± 16.1*	209.7 ± 14.1	237.7 ± 10.7
RR	56.5 ± 7.2	38 ± 8.6	32.3 ± 6.4	30.8 ± 5.3	29.6 ± 5.5	28.9 ± 3.7	26.4 ± 4.4	21.7 ± 4.1*
SABP	101.4 ± 4.9	88.1 ± 10.4	78.4 ± 17.1	66.5 ± 7.5	60.7 ± 14.2*	64.6 ± 16.4	48.7 ± 14.2*	47.4 ± 9.3*
DABP	71.9 ± 8.5	66.4 ± 8.7	55.7 ± 14.1	37.1 ± 10.2*	36.4 ± 9.7*	37.7 ± 8.6*	29.4 ± 4.5*	25.1 ± 5.6*
MABP	69.4 ± 10.8	64.7 ± 8.6	56.6 ± 12.2	40.1 ± 17.4	42.0 ± 13.1	39.7 ± 11.4*	36.7 ± 9.6*	34.0 ± 7.3*
SpO_2_	97.7 ± 2.5	98.2 ± 3.7	95,6 ± 3.9	94.4 ± 4.1	93.4 ± 4.7	90.1 ± 1.8	89.4 ± 1.4	86.3 ± 2.8
T °C	38.1 ± 0.6	38.3 ± 0.8	37.9 ± 1.2	37.9 ± 0.7	37.6 ± 0.8	37.8 ± 0.5	37.2 ± 0.6	37.2 ± 0.2
IoC	88.3 ± 1.1	81.9 ± 8.6	76.5 ± 5.2	70.6 ± 7.2	69.7 ± 5.5	67.3 ± 5.8	65.0 ± 4.1*	62.2 ± 4.3*

Mean ± SD, n = 7; * p < 0.05 when compared with time before anesthesia (0 h)

*PaCO*_*2*_ partial pressure of carbon dioxide, *PaO*_*2*_ partial pressure of oxygen, *SABP* systolic arterial blood pressure, *DABP* diastolic arterial blood pressure, *MABP* mean arterial blood pressure are expressed in mmHg, *cHCO*_*3*_^*−*^ bicarbonate ion concentration in mmol/L, *HR* heart rate in beats per minute, *RR* respiratory rate in movements per minute, *SpO*_*2*_ peripheral oxygen saturation in %, *T* temperature in °C, *IoC* index of consciousness

Concerning blood biochemistry, changes between blood parameters were observed at different time-points in the propofol Lipuro and SMOFlipid emulsion groups. In the saline group, no differences were found during the entire study. A large number of parameters (ALP, cholesterol, glucose, triglycerides, CK, Na^+^) exhibited statistical differences from T12 after the onset of propofol Lipuro and SMOFlipid administration till the end of the infusion. From a general overview of each group, the propofol Lipuro group was the one that showed more significant differences among the parameters in study during the 20 h protocol. Table 2 shows the comparison between groups made at specific time-points for blood biochemistry parameters, statistical differences are observed not only between the saline and the propofol Lipuro group but also between the saline and SMOFlipid and the SMOFlipid and the propofol Lipuro group. At the end (T20) the mean propofol concentration was 52.08 ± 15.65 μg mL^−1^ in plasma whereas in the liver was 2.98 ± 0.69 μg g^−1^.

**Table 2:** Blood biochemistry differences (Mean ± SD, n = 7) between the study groups (saline, SMOFlipid and propofol Lipuro) obtained from 12 to the 20 h of drug infusion

Parameter	Saline	SMOFlipid	Propofol Lipuro
	12 h sampling		
ALP	144.8 ± 27.0^a^	315.7 ± 32.6^b^	198.7 ± 46.6^c^
ALT	23,6 ± 9.6^a^	75.9 ± 25.7^b^	46.4 ± 15.5^c^
AST	20.8 ± 6.4^a^	68.8 ± 21.5^b^	38.3 ± 25.1^a^
Glucose	106.6 ± 15.1^a^	135.2 ± 24.4^b^	124.1 ± 14.5^a,b^
Cholesterol	29.0 ± 5.1^a^	73.1 ± 43.5^b^	106.6 ± 16.9^c^
Triglycerides	55.2 ± 12.8^a^	922.1 ± 194.3^b^	768.6 ± 124.7^b^
LDH	140.6 ± 33.7^a^	671.9 ± 358.8^b^	588.9 ± 285.2^b^
CK	1149.5 ± 261.3^a^	4898.0 ± 214.1^b^	1278.7 ± 368.8^c^
Amylase	302.8 ± 35.6^a^	374.6 ± 91.4^a^	449.9 ± 87.9^b^
Urea	28.4 ± 8.5^a^	26.4 ± 4.6^a^	69.5 ± 18.7^b^
Creatinine	0.9 ± 0.7^a^	1.0 ± 0.3^a^	2.9 ± 0.4^b^
Phosphorus	4.3 ± 1.0^a^	7.8 ± 3.2^b^	10.7 ± 3.8^b^
Ca2+	11.7 ± 0.8^a,b^	12.5 ± 3.5^a^	9.7 ± 1.2^b^
Na+	129.9 ± 12.1^a^	145.3 ± 12.3^b^	140.4 ± 5.9^a,b^
	15 h sampling		
ALP	148.0 ± 16.1^a^	255.4 ± 44.1^b^	171.8 ± 48.7^a^
ALT	27.0 ± 8.6^a^	65.6 ± 34.9^b^	45.3 ± 13.7^a^
AST	25.5 ± 4.8^a^	42.3 ± 27.1^a^	88.7 ± 29.1^b^
Glucose	105.5 ± 8.9^a^	134.3 ± 18.1^b^	126.1 ± 15.7^a,b^
Cholesterol	36.6 ± 6.4^a^	79.8 ± 37.4^b^	122.5 ± 14.8^c^
Triglycerides	61.8 ± 26.8^a^	1066.7 ± 203.1^b^	1022.7 ± 94.2^b^
LDH	142.8 ± 39.4^a^	730.3 ± 366.2^b^	587.7 ± 181.1^b^
CK	1122.8 ± 172.8^a^	7225.4 ± 863.7^b^	1840.0 ± 390.7^c^
Amylase	326.6 ± 51.1^a^	371.6 ± 88.2^a^	459.9 ± 101.6^b^
Urea	25.5 ± 7.1^a^	28.7 ± 7.1^a^	82.2 ± 13.3^b^
Creatinine	1.2 ± 0.4^a^	1.3 ± 0.3^a^	3.4 ± 0.5^b^
Phosphorus	4.2 ± 0.8^a^	8.6 ± 2.8^b^	11.8 ± 5.5^c^
Ca2+	11.8 ± 0.7^a^	1.4 ± 2.5^a^	9.7 ± 2.1^a^
Na+	139.2 ± 10.2^a^	144.2 ± 4.4^a^	140.6 ± 7.8^a^
	18 h sampling		
ALP	132.2 ± 21.8^a^	260.1 ± 49.8^b^	173.8 ± 44.2^a^
ALT	23.0 ± 12.9^a^	58.4 ± 22.1^b^	56.1 ± 15.8^a^
AST	19 ± 4.7^a^	33.0 ± 18.5^a^	100.4 ± 18.6^b^
Glucose	101.8 ± 12.2^a^	120.4 ± 26.1^a^	127.6 ± 20.8^a^
Cholesterol	30.7 ± 6.2^a^	82.1 ± 58.5^b^	131.3 ± 27.8^c^
Triglycerides	73.6 ± 26.9^a^	1608.5 ± 226.2^b^	1119.6 ± 112.3^c^
LDH	158.5 ± 51.2^a^	879.6 ± 373.7^b^	602.5 ± 331.4^c^
CK	1113.4 ± 468.6^a^	6584.5 ± 542.1^b^	2063.1 ± 495.5^c^
Amylase	369.5 ± 32.1^a^	586.2 ± 101.4^b^	513.7 ± 138.2^b^
Urea	26.4 ± 6.4^a^	18.9 ± 3.3^a^	95.3 ± 10.0^b^
Creatinine	0.9 ± 0.6^a^	1.2 ± 0.4^a^	3.7 ± 0.4^b^
Phosphorus	3.9 ± 0.6^a^	9.1 ± 3.2^b^	11.9 ± 7.4^b^
Ca2+	12.1 ± 1.9^a^	11.1 ± 3.1^a^	9.6 ± 1.9^a^
Na+	139.6 ± 9.5^a^	143.3 ± 9.5^a^	139.2 ± 6.9^a^
	20 h sampling		
ALP	135.8 ± 16.4^a^	273.8 ± 47.2^b^	189 ± 52.7^c^
ALT	25,3 ± 11.4^a^	73.6 ± 26.2^b^	48.0 ± 18.0^c^
AST	29.2 ± 13.2^a^	47.6 ± 17.4^a^	140.0 ± 24.2^b^
Glucose	99.8 ± 13.1^a^	147.6 ± 17.9^b^	125.8 ± 14.3^a,b^
Cholesterol	30.2 ± 7.6^a^	87.6 ± 57.4^b^	138.7 ± 48.9^c^
Triglycerides	66.1 ± 15.8^a^	1685.4 ± 103.4^b^	1086.8 ± 254.1^c^
LDH	155.2 ± 56.7^a^	750.0 ± 256.2^b^	607.0 ± 272.4^b^
CK	1035.8 ± 385.2^a^	10,492.0 ± 874.3^b^	3988.8 ± 657.6^c^
Amylase	336.2 ± 27.9^a^	668.9 ± 112.4^b^	502.7 ± 112.3^c^
Urea	26.7 ± 4.2^a^	23.2 ± 5.5^a^	89.7 ± 15.2^b^
Creatinine	1.03 ± 0.4^a^	1.1 ± 0.3^a^	3.9 ± 0.7^b^
Phosphorus	4.3 ± 0.9^a^	9.7 ± 4.6^b^	12.4 ± 3.1^b^
Ca2+	11.7 ± 1.7^a^	12.4 ± 4.0^a^	9.9 ± 2.6^a^
Na+	130.5 ± 14.2^a^	137.5 ± 5.4^a^	139.4 ± 5.8^a^

Different letters show significant differences between study groups (p < 0.05)

### Histopathological report

**Fig. 1:**
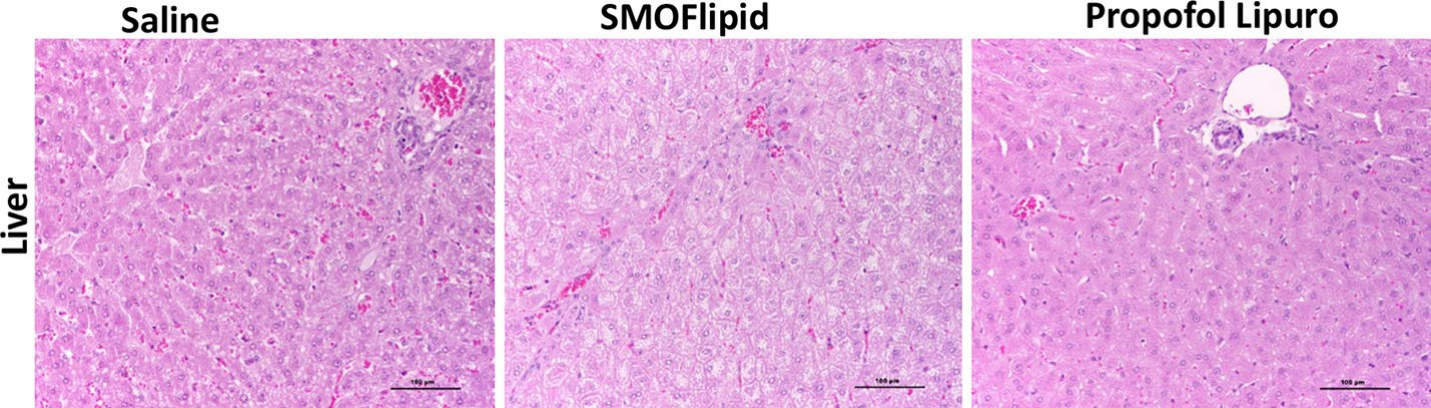
Microscopic images (H&E) of liver from animals of each study group (propofol Lipuro, SMOFlipid and saline) at the end of the 20 h of treatment. Liver changes in animals from the saline and propofol groups were similar and characterized by occasional hepatic cellular tumefaction or mild microvacuolar/hydropic degeneration. In the SMOFlipid group, hepatic lesions of massive hydropic degeneration, consistent with generalized steatosis. The majority of animals tested had no microscopic cardiac lesions, however myocardial vacuolar degeneration, focal necrosis and myocarditis were observed in the propofol group (*scale bar* 50 µm)

Histopathological results showed that microscopic cardiac lesions were absent in animals from saline and SMOFlipid groups, however, in the propofol Lipuro group, myocardial vacuolar degeneration, focal necrosis and myocarditis were observed. Occasional hepatic cellular tumefaction or mild microvacuolar/hydropic degeneration was present in animals from the saline and propofol Lipuro groups, which was focal and without pathological significance. In this last group, single cell necrosis, as well as multifocal hepatitis with heterophils and occasional lymphocytes were also present. The animals from the SMOFlipid group displayed hepatic lesions of massive hydropic degeneration, consistent with generalized steatosis (Fig. 1). Microscopic examination of the lungs from the propofol Lipuro and SMOFlipid groups revealed atelectasis and arterial vascular lesions, which included sub-endothelial vasculitis with heterophils and some eosinophils, intimal hyperplasia and degenerative changes of the muscle cells in the medial arterial wall. In some of the animals from the propofol Lipuro and SMOFlipid groups these vascular changes were accompanied by fragmentation of the internal elastic membrane. Animals from these two groups also presented sparse interstitial lymphocytic inflammation, as well as an increased number of alveolar macrophages. With the exception of animals from the propofol Lipuro group, which had microscopic changes consistent with proteinuria and acute renal failure, no significant renal changes were observed in the other groups (SMOFlipid and saline). Skeletal muscle and urinary bladder microscopic observation revealed no significant lesions in all the animals from the three groups under study.

### Effects on mitochondria respiratory chain function and mitochondrial coupling

**Fig. 2:**
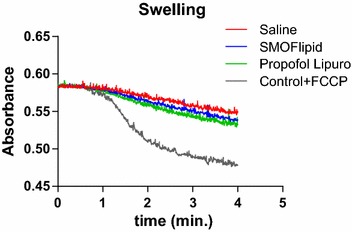
Mitochondrial swelling results observed in all three groups (propofol Lipuro, SMOFlipid and saline). The control experiment was made in the absence of carbonylcyanide p-trifluoromethoxyphenylhydrazone (FCCP) and propofol. Maximum swelling was obtained with FCCP (1 mM). Valinomycin (1 mM) was added after 1 min. *The traces* are representative of a group of at least three independent experiments

The respiratory activity of isolated liver mitochondria was measured using two different substrates, namely, pyruvate-malate and succinate and the results are presented in Table 3. The state 3 and 4, reflecting ADP-stimulated and ADP-absence respiration, respectively, showed no significant differences when complex I substrate was used. However, when using succinate as respiratory substrate, a distinct trend to decrease in ADP-stimulated respiration rate was observed for the SMOFlipid group (p = 0.0005) compared to control group. The coupling between respiration and phosphorylation (RCR) and the ratio of ADP molecules phosphorylated to oxygen atoms reduced (ADP/O) was not significantly affected in all groups when both substrates were used. Also, no differences were observed in the mitochondrial membrane potential when succinate was used, indicating that respiratory coupling and electron transport chain function were not significantly affected. Furthermore, no statistical differences between groups were observed in mitochondrial swelling, which was confirmed using the oxidative phosphorylation uncoupler FCCP (Fig. 2).

**Table 3:** Results from respiratory chain activity when different substrates obtained from different mitochondrial preparations for each experimental group energized with pyruvate-malate or succinate (5 mM)

Parameter	Group			Statistical test	*p*
	Saline	SMOFlipid	Propofol Lipuro		
Pyruvate-malate substrate					
State 3 rate (nmol O_2_ min^−1^ mg protein^−1^)	54.66 ± 16.97	47.81 ± 18.04	41.07 ± 15.04	F(2,17) = 1.67	0.34
State 4 rate (nmol O_2_ min^−1^ mg protein^−1^)	16.82 [13.58–19.60]	20.06 [14.24–28.35]	15.48 [11.23–20.77]	χ^2^(2) = 1.29	0.52
Uncoupled rate (nmol O_2_ min^−1^ mg protein^−1^)	44.59 ± 17.08	47.99 ± 18.36	50.25 ± 17.99	F(2,17) = 0.18	0.84
RCR	2.48 [2.20–3.11]	2.34 [1.92–2.91]	2.81 [2.34–3.12]	χ^2^(2) = 3.08	0.21
ADP/O	2.60 ± [2.34–2.94]	2.85 [2.79–2.91]	2.64 [2.37–2.97]	χ^2^(2) = 0.72	0.69
Succinate substrate					
State 3 rate (nmol O_2_ min^−1^ mg protein^−1^)	51.68 ± 3.42^a^	38.42 ± 4.15^b^	45.66 ± 7.29^a,b^	F(2,18) = 9.13	0.002
State 4 rate (nmol O_2_ min^−1^ mg protein^−1^)	22.99 ± 4.26	20.63 ± 5.83	21.34 ± 3.37	F(2,20) = 2.84	0.09
Uncoupled rate (nmol O_2_ min^−1^ mg protein^−1^)	177.5 ± 39.4	141.8 ± 35.47	166.8 ± 23.06	F(2,18) = 3.05	0.08
RCR ou ICR	2.40 ± 0.17	2.06 ± 0.10	2.16 ± 0.12	F(2,18) = 1.59	0.23
ADP/O	1.34 ± 0.40	1.33 ± 0.39	1.33 ± 0.35	F(2,19) = 0.15	0.86
Membrane potential	171.7 ± 30.15	145.8 ± 37.5	156.8 ± 26.1	F(2,20) = 1.18	0.33

Results expressed by M ± SD (mean ± standard deviation) or median and interquartile range for liver mitochondria (1 mg mL^−1^ of protein). Oxidative phosphorylation was measured polarographically at 25 °C in 1 mL. ΔΨ was measured using a TPP^+^-selective electrode at 25 °C in a total volume of 1 mL. Different letters show significant differences between groups (p < 0.05)

### Effects on mitochondria respiratory chain complexes activities

The enzymatic activity of the mitochondrial respiratory chain complexes I, I–III, II, II–III, III and IV were determined by spectrophotometric methods and normalized to the citrate synthase activity, which was similar between groups, and are displayed in Table 4. Despite a slight decrease, but not statistically significant (30 %), in the enzyme activity of complex IV in the propofol Lipuro group, no other significant differences in the specific activities of all other mitochondrial complexes were found.

**Table 4:** Propofol effects on liver mitochondrial respiratory chain activities after the 20 h infusion in the 3 groups

Activity	Group			Statistical test	*p*
	Saline	SMOFlipid	Propofol Lipuro		
Complex I	4.43 ± 2.33	4.08 ± 0.98	3.60 ± 1.54	F(2,17) = 0.35	0.71
Complex I–III	13.63 ± 5.76	14.44 ± 2.29	13.07 ± 4.32	F(2,17) = 0.15	0.86
Complex II	0.19 ± 0.11	0.14 ± 0.08	0.25 ± 0.11	F(2,17) = 0.68	0.52
Complex II–III	5.01 ± 2.33	4.14 ± 1.54	4.54 ± 1.96	χ^2^(2) = 0.13	0.94
Complex III	2.11 ± 1.64	2.06 ± 0.57	1.68 ± 0.71	χ^2^(2) = 0.72	0.69
Complex IV	14.81 ± 9.48	16.34 ± 7.56	10.25 ± 7.79	χ^2^(2) = 5.54	0.06
Citrate synthase	2.26 ± 1.09	1.56 ± 0.34	2.10 ± 0.63	F(2,17) = 1.43	0.27

Results expressed by M ± SD (mean ± standard deviation) or median and interquartile range for liver mitochondria (10 μg of protein) obtained from different mitochondrial preparations for each experimental group. No statistical significance was observed between groups

### Effects of propofol on oxidative stress

**Fig. 3:**
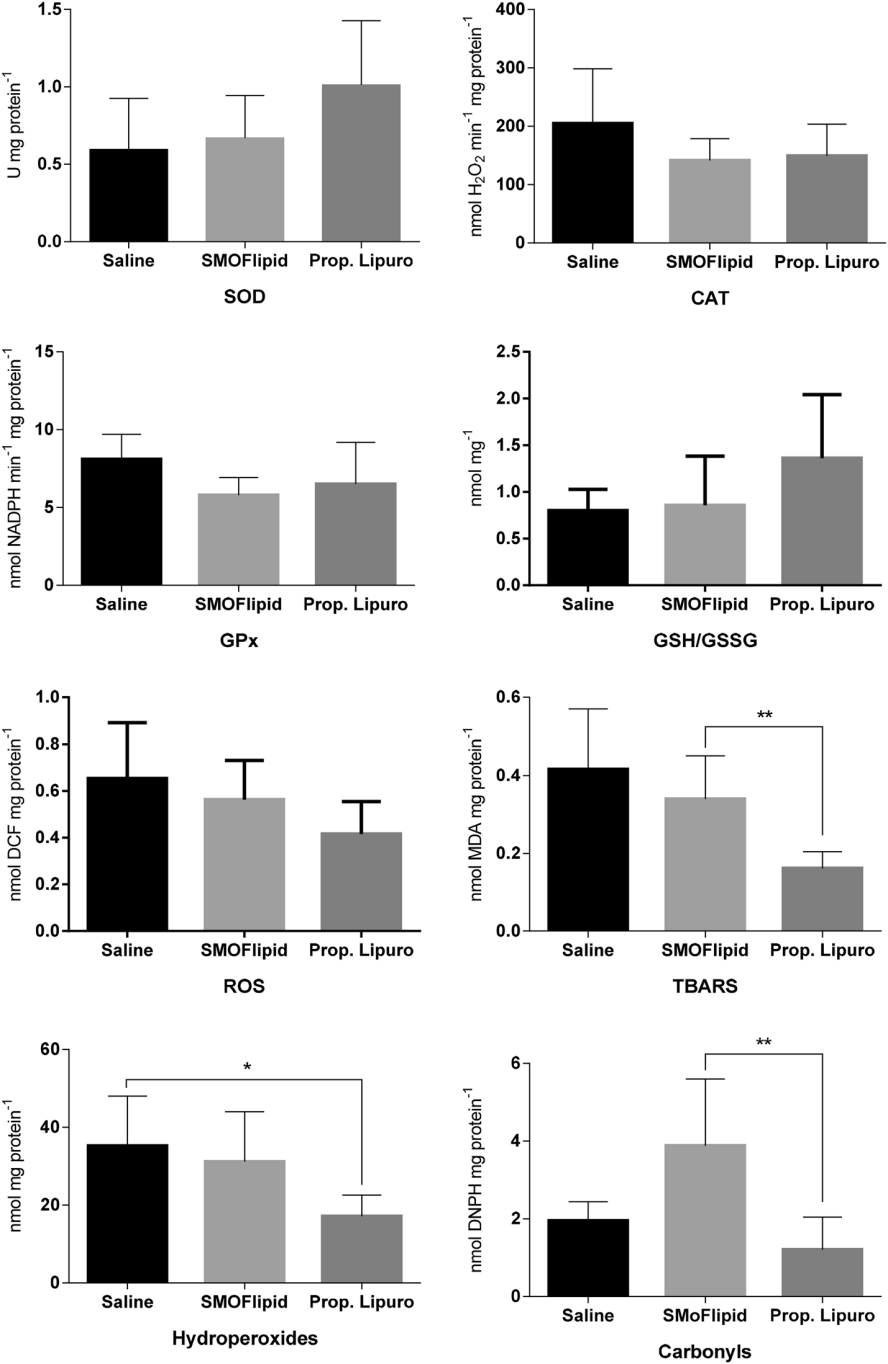
Effects of Propofol Lipuro and the SMOFlipid emulsion on the activities of enzymatic and non-enzymatic antioxidants obtained from different mitochondrial preparations for each experimental group, at the end of the infusion time (T20). *Asterisks* indicates statistical significance between study groups (p < 0.05)

Figure 3 shows the effects of propofol Lipuro and SMOFlipid emulsion on the activities of enzymatic and non-enzymatic antioxidants. Despite a slight increase in SOD activity for SMOFlipid and propofol Lipuro groups, no statistical significant differences were observed between groups. Similarly, the analysis of CAT and GPx showed no significant differences between groups, even though lower values were observed for the SMOFlipid and propofol Lipuro groups. The redox status of the cell, measured through the GSH/GSSHG ratio, showed no statistically significant tendency to increase with the administration of propofol Lipuro and the SMOFlipid emulsion. Lipid peroxides showed a statistically significant difference between propofol Lipuro and saline (p < 0.0001) and SMOFlipid (p < 0.0001) groups. A similar profile was observed for propofol hydroperoxides (p = 0.03) when compared to the saline control group. Between the SMOFlipid and control group no differences were observed. Protein carbonyls only reflected a significant difference between propofol Lipuro and the SMOFlipid (p = 0.009) group. The effect of propofol Lipuro and SMOFlipid treatment on ROS production was also evaluated and no statistical differences were observed between groups, despite a slight decrease (37 %) for propofol Lipuro group.

## Discussion

Propofol infusion syndrome (PRIS) has been extensively described in the last decade in humans, mostly in critically ill children and intensive care adult patients undergoing long-term sedation at high doses of propofol (Fodale and La Monaca 2008; Kam and Cardone 2007; Ahlen et al. 2006; Vasile et al. 2003; Fudickar and Bein 2009). In vivo studies were conducted by Yspilantis et al. (2007), where New Zealand rabbits were continuously sedated for 32.3 ± 5.4 h with propofol and after 20 h of anesthesia they showed blood biochemical and histological changes in the main organs resembling PRIS (Ypsilantis et al. 2007). In our study, all animals survived the 20 h anesthesia with a mean infusion rate of propofol was 37.6 ± 18.1 mg kg^−1^ h^−1^. Clinical and hemodynamic changes were noticed 12 h after the propofol infusion start. Sedated animals showed progressive variations in clinical parameters and blood biomarkers during the monitoring period, conducting to a characteristic set of signs that resemble human PRIS. Although the doses used in our study were high comparatively to that used in humans, the doses used were just to maintain the animals in a sedated level of anesthesia, as it was showed by the IoC values, that were never lower than 60, indicating that no overdoses were reached. Ypsilantis and colleagues (2007) anesthetized three groups of rabbits: one continuously infused with propofol (initial infusion rate of 20 mg kg^−1^ h^−1^ increased up to 65.7 mg kg^−1^ h^−1^ for maximum period of 38 h where animals died by themselves), another with sevoflurane (ranging from 1.5–4 %) and one last with sevoflurane and intravenous Intralipid 10 %, all under mechanical ventilation. The amount of Intralipid delivered to animals was not specified in the Ypsilantis’ study. Changes in hepatic biomarkers and histologic lesions in liver, kidneys and lungs were observed in the propofol and Intralipid groups, indicating that propofol lipid emulsion can also be responsible for part of this syndrome (PRIS) (Ypsilantis et al. 2007). In our study, we evaluated the effects of a 20 h prolonged high-dose administration of propofol Lipuro and an improved lipid emulsion as SMOFlipid, separately administered to rabbits (Adolph et al. 2009), on liver mitochondrial bioenergetics and oxidative stress without the use of other concomitant anesthetics, without the presence of confounding variables, as other anesthetics.

Clinical signs monitored in the propofol Lipuro group showed bradycardia followed by reflexive tachycardia due to hypotension. Progressive decreases in ${\text{HCO}}_{3}^{ - }$ concentration in arterial blood and pH suggest the development of metabolic acidosis mainly due to hypoxia and hypoperfusion with progressive failure of the heart and kidneys caused by propofol prolonged infusion and its accumulation in tissues (Ypsilantis et al. 2007; Campos et al. 2015). Although the lactate values were not monitored, the increase of the Cl^−^ concentration in the final hours seems to compensate the low ${\text{HCO}}_{3}^{ - }$ (Fudickar and Bein 2009). Several studies suggest that the mechanisms of hypotension are propofol-mediated decreases in sympathetic activity including decrease in cardiac output due to venous and arterial vasodilatation, impaired baroreflex mechanism and depression of myocardial contractility (Robinson et al. 1997). Our study showed that the sympathoinhibitory effects of propofol particularly observed near the end of the study by the severe cardiovascular depression, contributed importantly to the hemodynamic consequences of propofol Lipuro anesthesia and driven to the results obtained for mitochondrial function and oxidative stress. This can be associated to an accumulation of propofol during prolonged infusions caused by the saturation of the metabolic enzymatic system (Campos et al. 2015). Indeed, according to, Chang et al. 2001 a significant decreased membrane potential and distorted mitochondria morphology of aortic endothelial cells mean that the clinical hypotension induced by propofol might be a potential mechanism (Chang et al. 2001). Histological analysis confirmed the presence of multi-organic lesions consistent with PRIS as: liver failure and steatosis, heart inflammation and renal failure (Ypsilantis et al. 2007; Fudickar and Bein 2009). These results together with changes observed in clinical signs, gas measurements and blood biomarkers (liver, renal and lipid ones), suggest that the animals under propofol Lipuro sedation developed signs compatible with PRIS after 20 h of infusion. Moreover, some clinical parameters in the SMOFlipid group such as serum lipids, liver enzymes, CK and sodium, revealed to be statistically different not only from the saline but also from the propofol Lipuro group while biomarkers as urea, creatinine and amylase did not change along time in the SMOFlipid group, evidencing that these animals did not develop renal or pancreatic lesions. The increase of the CK levels may result from heart and renal failure/inflammation and/or incipient rhabdomyolysis. Similar changes in cardiac function during anesthesia have been described in human patients with PRIS (Kam and Cardone 2007; Bray 1998). A progressive bradycardia was noticed until 15 h of propofol Lipuro infusion followed by evident tachycardia, possibly reflex to hypotension observed after this period (Martin-Cancho et al. 2006). Histologic examination revealed myocarditis and smooth degeneration consistent with myofibril degeneration or rhabdomyolysis, however, the absence of ECG monitoring did not allow the identification of asystole in animals around the 20 h of anesthesia. Kidney biomarkers in blood (urea, creatinine, CK) were above the maximum limit, indicating late injury and failure. This was also confirmed by microscopy that revealed nephritis and proteinuria. Long-term propofol administration is also related with variations in pancreatic enzymes, especially in children. The high levels of amylase in the propofol group in end of the study, together with the high levels of triglycerides, suggests an early pancreatic inflammation (Gottschling et al. 2005) but no histologic examination was performed to the pancreas.

Hepatomegaly, hypertriglyceridemia and cholestatic liver have been associated with high lipid load and therapeutic failure during long-term propofol administration (Adolph et al. 2009; Knibbe et al. 2004) and were observed in the propofol Lipuro and the SMOFlipid groups. Fatty acids are metabolized in many tissues including hepatocytes, myocardium and muscles. Clinical, in vitro and in vivo studies pointed that lipid infusions may induce not only immunosuppressive effects (Adolph et al. 2009; Calder et al. 2002) but also impaired fat metabolism (Lindholm 1992). A continuous lipid administration that exceeds the rate of oxidation can evolve to a ‘Fat overload syndrome’ (FOS). Excess lipid particles are up taken by immune cells (mononuclear phagocytes generating immunodeficiency, known by systemic inflammatory response syndrome (SIRS) (Adolph et al. 2009; Palmblad 1991). Curiously, FOS, SIRS and PRIS have similar clinical manifestations: hepatomegaly, increased serum lipids, metabolic acidosis among others. In addition, cells membranes contain fatty acids that can affect numerous membrane properties as fluidity, transport processes, the activity of membrane proteins and receptors and also modify gene expression by binding to nuclear receptors (Adolph et al. 2009). While propofol formulations are prepared in lipid vehicles, its use during long-term anesthesia and sedation, increases the probability of PRIS occurrence. PRIS results from side-effects of propofol over non-targeted organs (like heart, lungs and kidneys) associated to prolonged lipid emulsion infusions. This may be also potentiated by the genetic susceptibility of each individual to metabolize xenobiotics (Kneiseler et al. 2010). The non-monitoring of serum lipids in patients receiving lipid-containing propofol infusions, frequently leads to an excess of triglycerides which can be a limiting factor for propofol dosing and therapeutic management (Knibbe et al. 2004; Kobayashi et al. 2008). In our study, the lipid formulations used (propofol Lipuro vs SMOFlipid emulsion) had different types of triglycerides (soya-bean vs. fish-oil based). SMOFlipid emulsion was chosen for lipid infusion due to its improved composition (by containing other oils than soya-bean) and lower incidence of side effects (multi-organ damage) compared with other vehicles (Koller et al. 2003), even so, many undesired changes were observed in our study. Notwithstanding, the clinical and scientific popularity achieved with parenteral nutrition or propofol emulsions challenged the discovery of new lipid-based formulations (Knibbe et al. 2004; Jung et al. 2010).

Several studies describe the pathophysiology of PRIS as an imbalance of the mitochondrial respiratory chain, leading to disruption on cellular energy production and, consequently, compromising Krebs cycle, metabolism of amino acids and fatty acid oxidation and consecutive cell death (Vasile et al. 2003; Kneiseler et al. 2010; Muravchick and Levy 2006). In fact, many in vitro and in vivo studies have reported relevant injury to the heart (Argaud et al. 2008; Shao et al. 2008), brain (Kiebish et al. 2008; Marian et al. 1997) and liver (Branca et al. 1991a, b; Rigoulet et al. 1996) mitochondria as a consequence of high propofol concentrations resulting in an impairment oxygen utilization and ATP production and decreased electron flow (Schenkman and Yan 2000; Muravchick and Levy 2006; Marian et al. 1997). Propofol also affects mitochondrial at complex I level as well as uncouples oxidative phosphorylation. It is also thought to interfere with complex II (Wolf et al. 2001), however, few studies have focused on the in vivo mitochondrial effects of propofol. In our work, we have determined respiratory function of liver mitochondria. Surprisingly, when using succinate as respiratory substrate for complex II, a significant decrease in ADP-stimulated respiration rate was observed for the SMOFlipid group, suggesting that the lipid emulsion inhibited the state-3 oxygen consumption rate, corresponding to the mitochondrial capacity to synthetize ATP, and revealing poor efficiency of proton-pumping activity and consequent decrease of the respiratory rate from this electron donor. This effect was observed in isolated rat liver mitochondria, when a subclinical dose of propofol was added to in vitro to a specific amount of isolation medium (Rigoulet et al. 1996). However, and despite the observed result for state-3 respiration, the analysis of the mitochondrial membrane potential showed no significant effects despite a slight decrease for the SMOFlipid group. The same was confirmed by the analysis of mitochondrial swelling indicating that the mitochondrial membrane was not affected by the treatments and thus preserving the mitochondrial function conversely to what was found by Wu et al. (2005). In fact, this is not the first time that this effect is documented for propofol (Adembri et al. 2006).

Additionally, the mitochondrial inner membrane lipid composition is of importance for the stabilization of the respiratory chain complexes (Zigdon et al. 2013) within which complex I and III have been identified as the major sources of ROS. In this study, contrarily to what was found by Vanlander et al. (2014), no significant differences were observed neither for succinate cytochrome *c* reductase (complex II + III) nor for complex II as observed by Kajimoto (2014). In fact, we have only observed a tendency to decrease complex IV activity which is regulated by the mitochondrial electric membrane potential modulating the activity of the ETC according to its needs (Desler et al. 2012). The importance of this complex for the optimal activities of complex I and III has been previously demonstrated (Schafer et al. 2006). Furthermore, as complex IV is particularly sensitive to changes in the fluidity of the membrane lipid composition (Trivedi et al. 1986), we can assume that our results are most likely due to propofol interaction with the membrane. In fact, it has been already described the ability of propofol to interact and change the membrane fluidity at clinically relevant concentrations (Bahri et al. 2007). This could be explained by an incorporation of propofol into the inner mitochondrial membrane and prevention of the opening of the MPTP which was already been observed in other studies (Adembri et al. 2006; Shirakawa et al. 2014).

Many studies have already showed the antioxidant capacity of propofol (Mathy-Hartert et al. 1998) which may be resultant from its α-tocopherol similar structure. In our study, we have analyzed several oxidative stress markers that could be affected by propofol Lipuro infusion and observed a significant decrease for lipid peroxides, carbonyls and hydroperoxides, which are the oxidative markers that are usually elevated as a result of toxic effect of drugs (Evans et al. 2002). The decrease observed in these parameters for this propofol formulation might be due to the antioxidative nature of propofol which could also contribute to the effects observed in the antioxidant enzymes (SOD, CAT and GPx) as already seen by Ranjbar et al. (2014). Regarding the SMOFlipid group, we found differences in protein carbonyls oxidation compared to the control group which can lead to changes in the physical properties of the proteins and consequently to the organ function and recent clinical studies have linked soybean-oil lipid emulsions to the occurrence of cholestasis (Kaffe et al. 2015). In fact, it was also showed that accumulation of free radicals in cholestatic liver disease induces carbonylated protein increments (Sokolovic et al. 2013) and thus, we hypothesize that SMOFlipid emulsion can result in this condition, especially in patients with associated liver impairment.

In conclusion, at the measured concentrations (2.98 ± 0.69 μg g^−1^) in the liver, propofol Lipuro formulation evidenced to have higher antioxidant activity and lower impairment of the mitochondrial function comparatively to the improved lipid formulation, SMOFlipid, using the rabbit as animal model, however, it also has collateral cardiorespiratory effects, by diminishing the metabolic rate, blood perfusion and oxygen delivery to the brain and vital organs which is consistent with the high propofol dose quantified in blood (52.08 ± 15.65 μg mL^−1^). As a final point, long-term high doses infusion of propofol Lipuro emulsion for sedation prompted changes in clinical and hemodynamic signs, particularly hypotension and bradycardia, blood gas levels and many blood biomarkers, as CK and serum lipids, liver and kidney parameters. At the end of the experiment, the animals under propofol Lipuro anesthesia showed also several organ failures that were compatible with PRIS. Many similar variations in blood biomarkers and multi-organ deterioration were observed in animals receiving the SMOFlipid (lipid emulsion) without the propofol on it. Mitochondrial bioenergetics results from this, long-term high dose infusion protocol of propofol Lipuro, indicate that mitochondria may not be the main and unique trigger compound of PRIS but we cannot exclude its involvement of other pathways in liver failure such as cell death via *apoptosis* and *necrosis* which were not addressed in this work. The contribution of hypotension and hypoperfusion for the final results cannot be discarded. Moreover, lipid emulsions may also play a role in the initial installation of the syndrome. We strongly recommend the close monitoring not only of patients and animals undergoing propofol Lipuro lipid-based prolonged sedation, but also receiving improved lipid emulsions as SMOFlipid for prolonged parenteral nutrition. These results may provide new strategies and approaches for the insight of the biochemical mechanisms underlying PRIS, using the rabbit as a translational model.

## Additional file

10.1186/s40064-016-2970-2 Blood biochemistry measurements (Mean ± SD, n=7) in saline, SMOFlipid and propofol Lipuro group during the 20h of drug infusion (*p<0.05 indicates statistical differences of time point when compared with T0 values).
