# Supplementary material for: In vivo study of hepatic oxidative stress and mitochondrial function in rabbits with severe hypotension after propofol prolonged infusion
Source: Springerplus. 2016 Aug 15;5(1):1349. doi: 10.1186/s40064-016-2970-2 (PMC4987748; doi:10.1186/s40064-016-2970-2)
Supplement: Supplementary file 1 — 10.1186/s40064-016-2970-2 Blood biochemistry measurements (Mean ± SD, n=7) in saline, SMOFlipid and propofol Lipuro group during the 20h of drug infusion (*p<0.05 indicates statistical differences of time point when compared with T0 values). [file 40064_2016_2970_MOESM1_ESM.docx]

**Table S1-** Blood biochemistry measurements (Mean ± SD, n=7) in saline, SMOFlipid and propofol Lipuro group during the 20h of drug infusion (*p<0.05 indicates statistical differences of time point when compared with T0 values).

|  | **Saline Group** - Time-points (h) | | | | | | | |
| --- | --- | --- | --- | --- | --- | --- | --- | --- |
| **Parameter** | **T0** | **T3** | **T6** | **T9** | **T12** | **T15** | **T18** | **T20** |
| **ALP** | 110.4±23.3 | 133.7±23.9 | 142.5±20.8 | 139.3±19.3 | 144.8±27.0 | 148.0±16.1 | 132.2±21.8 | 135.8±16.4 |
| **ALT** | 29,7±13.6 | 23,8±3.7 | 22,2±7.8 | 22,7±7.2 | 23,6±9.6 | 27.0±8.6 | 23.0±12.9 | 25,3±11.4 |
| **AST** | 16.7±6.7 | 23.5±11.4 | 20.8±7.9 | 26±10.9 | 20.8±6.4 | 25.5±4.8 | 19±4.7 | 29.2±13.2 |
| **TB** | 0.3±0.1 | 0.2±0.1 | 0.3±0.1 | 0.3±0.2 | 0.4±0.2 | 0.3±0.1 | 0.3±0.2 | 0.4±0.2 |
| **DB** | 0.1±0.05 | 0.07±0.04 | 0.1±0.06 | 0.1±0.06 | 0.1±0.06 | 0.1±0.08 | 0.1±0.07 | 0.1±0.04 |
| **Cholesterol** | 22.0±6.1 | 37.3±11.6 | 30.8±9.2 | 25.0±4.9 | 29.0±5.1 | 36.6±6.4 | 30.7±6.2 | 30.2±7.6 |
| **Triglycerides** | 62.3±13.2 | 41.8±6.8 | 46.5±15.1 | 51.5±17.1 | 55.2±12.8 | 61.8±26.8 | 73.6±26.9 | 66.1±15.8 |
| **LDH** | 113.2±29.4 | 137.2±31.9 | 139.8±41.7 | 134.5±43.8 | 140.6±33.7 | 142.8±39.4 | 158.5±51.2 | 155.2±56.7 |
| **Glucose** | 91.0±12.7 | 90.2±12.6 | 107.8±10.2 | 95.8±12.5 | 106.6±15.1 | 105.5±8.9 | 101.8±12.2 | 99.8±13.1 |
| **TP** | 6.2±1.0 | 5.1±0.9 | 5.8±0.9 | 4.9±1.7 | 4.6±1.1 | 4.2±0.8 | 4.5±0.6 | 5.0±1.6 |
| **Creatinine** | 0.86±0.2 | 1.0±0.3 | 1.1±0.3 | 1.2±0.5 | 0.9±0.7 | 1.2±0.4 | 0.9±0.6 | 1.03±0.4 |
| **Urea** | 26.2±6.4 | 30.5±6.3 | 25.2±13.5 | 25.1±6.2 | 28.4±8.5 | 25.5±7.1 | 26.4±6.4 | 26.7±4.2 |
| **CK** | 1093.4±120.0 | 981.2±203.1 | 1000.7±183.3 | 1081.5±172.8 | 1149.5±261.3 | 1122.8±172.8 | 1113.4±468.6 | 1035.8±385.2 |
| **amylase** | 330.7±65.6 | 388.7±43.2 | 339.3±59.4 | 346.2±49.7 | 302.8±35.6 | 326.6±51.1 | 369.5±32.1 | 336.2±27.9 |
| **Ca^2+^** | 11.9±3.6 | 11.7±1.2 | 11.9±1.2 | 10.9±1.1 | 11.7±0.8 | 11.8±0.7 | 12.1±1.9 | 11.7±1.7 |
| **Na^+^** | 125.6±10.8 | 126.6±14.2 | 132.7±10.4 | 119.9±14.6 | 129.9±12.1 | 139.2±10.2 | 139.6±9.5 | 130.5±14.2 |
| **K^+^** | 4.6±0.9 | 4.1±0.7 | 3.3±0.6 | 3.7±0.5 | 3.4±0.4 | 3.0±0.5 | 3.2±0.6 | 3.6±1.1 |
| **Cl^-^** | 108.9±7.9 | 107.4±9.9 | 112.8±6.4 | 115.0±10.2 | 119.3±8.8 | 125.5±13.0 | 112.7±3.8 | 114.5±2.7 |
| **Mg^2+^** | 2.5±1.0 | 3.3±1.1 | 3.3±1.2 | 3.1±0.8 | 2.9±0.8 | 2.6±0.7 | 2.1±1.1 | 2.8±1.6 |
| **Phosphorus** | 4.1±1.1 | 4.8±0.7 | 4.8±0.8 | 3.8±0.7 | 4.3±1.0 | 4.2±0.8 | 3.9±0.6 | 4.3±0.9 |
| **Hematocrit** | 38±2.3 | 38±1.7 | 37±2.1 | 37±1.6 | 37±1.3 | 37±1.1 | 36±0.8 | 36±0.9 |
|  | **SMOFlipid Group** | | | | | | | |
| **ALP** | 119.8±20.4 | 196.0±25.3* | 238.2±56.9* | 223.8±43.9* | 315.7±32.6* | 255.4±44.1* | 260.1±49.8* | 273.8±47.2* |
| **ALT** | 29.6±8.5 | 33.3±9.8 | 41.7±10.1 | 50.0±13.7 | 75.9±25.7* | 65.6±34.9* | 58.4±22.1* | 73.6±26.2* |
| **AST** | 13.9±5.7 | 20.8±11.7 | 43.3±22.9* | 52.2±24.4* | 68.8±21.5* | 42.3±27.1* | 33.0±18.5 | 47.6±17.4* |
| **TB** | 0.4±0.1 | 0.5±0.2 | 0.5±0.2 | 0.7±0.3 | 1.3±0.3* | 1.5±0.5* | 2.3±0.5* | 2.5±0.6* |
| **DB** | 0.09±0.05 | 0.11±0.04 | 0.12±0.06 | 0.15±0.51 | 0.30±0.14 | 0.35±0.19* | 0.53±0.21* | 0.67±0.26* |
| **Cholesterol** | 23.1±8.7 | 44.0±14.1 | 55.0±14.9 | 65.3±9.5* | 73.1±43.5* | 79.8±37.4* | 82.1±58.5* | 87.6±57.4* |
| **Triglycerides** | 51.0±10.9 | 622.5±178.4* | 693.7±164.7* | 730.8±189.3* | 922.1±194.3* | 1066.7±203.1* | 1608.5±226.2* | 1685.4±103.4* |
| **LDH** | 92.1±37.4 | 616.4±298.5* | 630.7±152.4 | 625.4±312.4 | 671.9±358.8 | 730.3±366.2 | 879.6±373.7* | 750.0±256.2* |
| **Glucose** | 82.4±11.1 | 96.5±45.8 | 121.4±51.9* | 111.0±31.8 | 135.2±24.4* | 134.3±18.1* | 120.4±26.1* | 147.6±17.9* |
| **TP** | 5.5±0.7 | 5.8±1.4 | 6.5±1.2 | 5.7±2.1 | 6.6±1.5 | 6.5±1.8 | 7.4±1.7 | 8.5±2.5* |
| **Creatinine** | 0.9±0.2 | 0.8±0.3 | 1.1±0.2 | 1.0±0.2 | 1.0±0.3 | 1.3±0.3 | 1.2±0.4 | 1.1±0.3 |
| **Urea** | 23.3±4.1 | 24.7±9.3 | 28.8±5.1 | 24.6±6.1 | 26.4±4.6 | 28.7±7.1 | 18.9±3.3 | 23.2±5.5 |
| **CK** | 875.3±207.9 | 2499.5±471.2* | 3336.3±291.5* | 4335.0±446.5* | 4898.0±214.1* | 7225.4±863.7* | 6584.5±542.1* | 10492.0±874.3* |
| **Amylase** | 294.2±93.3 | 293.6±80.2 | 339.3±47.5 | 353.0±63.7 | 374.6±91.4 | 371.6±88.2 | 586.2±101.4 | 668.9±112.4 |
| **Ca^2+^** | 12.6±1.4 | 11.1±0.6 | 11.2±2.3 | 9.9±1.7 | 12.5±3.5 | 1.4±2.5 | 11.1±3.1 | 12.4±4.0 |
| **Na^+^** | 115.5±5.5 | 137.3±4..3* | 142.2±9.5* | 143.1±6.9* | 145.3±12.3* | 144.2±4.4* | 143.3±9.5* | 137.5±5.4* |
| **K^+^** | 3.8±2.1 | 8.5±1.1 | 6.8±5.9 | 8.4±4.3* | 4.7±3.9* | 4.1±3.2* | 3.7±2.3* | 3.3±4.9* |
| **Cl^-^** | 110.2±8.1 | 116.5±5.0 | 119.2±5.9 | 121.3±4.3 | 117.8±6.9 | 117.3±4.2 | 115.6±7.3 | 112.4±4.8 |
| **Mg^2+^** | 2.1±0.5 | 2.0±1.0 | 2.8±0.5 | 2.3±1.2 | 2.8±0.7 | 3.7±1.2* | 2.1±1.2 | 2.5±0.5 |
| **Phosphorus** | 4.7±0.7 | 4.8±0.5 | 5.3±2.3 | 6.9±0.9 | 7.8±3.2 | 8.6±2.8 | 9.1±3.2 | 9.7±4.6* |
| **Hematocrit** | 41.8±3.1 | 41.5±2.9 | 40.1±2.3 | 39.4±2.5 | 38.2±2.6 | 38.0±1.4 | 37.3±1.1* | 37.1±0.9* |
|  | **Propofol Lipuro Group** | | | | | | | |
| **ALP** | 123.8±21.2 | 256.1±27.4* | 209.4±42.6* | 213.5±59.0* | 198.7±46.6* | 171.8±48.7 | 173.8±44.2 | 189±52.7* |
| **ALT** | 32.6±12.0 | 32.7±4.9 | 33.4±9.7 | 34.0±10.8 | 46.4±15.5 | 45.3±13.7 | 56.1±15.8 | 48.0±18.0 |
| **AST** | 17.6±6.0 | 23.3±10.3 | 20.7±12.5 | 34.7±20.3 | 38.3±25.1 | 88.7±29.1* | 100.4±18.6* | 140.0±24.2* |
| **TB** | 0.3±0.2 | 0.4±0.1 | 0.5±0.2 | 0.8±0.3 | 0.9±0.5* | 1.4±0.4* | 1.5±0.3* | 1.9±0.3* |
| **DB** | 0.08±0.03 | 0.1±0.05 | 0.1±0.07 | 0.2±0.1 | 0.2±0.1 | 0.3±0.1* | 0.4±0.1* | 0.5±0.2* |
| **Cholesterol** | 36.3±9.4 | 57.7±15.9 | 76.0±18.5 | 97.0±12.6* | 106.6±16.9* | 122.5±14.8* | 131.3±27.8* | 138.7±48.9* |
| **Triglycerides** | 75.0±18.7 | 531.6±98.4* | 641.9±112.5* | 703.3±108.8* | 768.6±124.7* | 1022.7±94.2* | 1119.6±112.3* | 1086.8±254.1* |
| **LDH** | 118.4±21.2 | 310.4±128.9 | 406.9±244.3 | 435.0±207.3 | 588.9±285.2* | 587.7±181.1* | 602.5±331.4* | 607.0±272.4* |
| **Glucose** | 84.9±22.6 | 112.7±12.4 | 122.3±11.3 | 124.7±15.1 | 124.1±14.5* | 126.1±15.7* | 127.6±20.8* | 125.8±14.3* |
| **TP** | 5.9±0.8 | 5.7±1.1 | 5.4±0.9 | 5.3±1.2 | 5.2±1.2 | 5.0±2.1 | 4.7±1.6 | 4.9±0.7 |
| **Creatinine** | 1.1±0.2 | 1.2±0.3 | 1.6±0.3 | 2.2±0.4* | 2.9±0.4* | 3.4±0.5* | 3.7±0.4* | 3.9±0.7* |
| **Urea** | 28.4±8.5 | 43.4±3.2* | 47.6±11.1* | 55.9±14.6* | 69.5±18.7* | 82.2±13.3* | 95.3±10.0* | 89.7±15.2* |
| **CK** | 705.5±128.1 | 1527.3±432.5* | 1307.9±321.8* | 1110.8±470.3* | 1278.7±368.8* | 1840.0±390.7* | 2063.1±495.5* | 3988.8±657.6* |
| **Amylase** | 317.9±84.2 | 344.8±68.1 | 354.3±61.9 | 400.2±75.3 | 449.9±87.9* | 459.9±101.6* | 513.7±138.2* | 502.7±112.3* |
| **Ca^2+^** | 14.5±2.3 | 10.8±1.9* | 10.2±2.5* | 9.9±2.0* | 9.7±1.2* | 9.7±2.1* | 9.6±1.9* | 9.9±2.6* |
| **Na^+^** | 110.6±7.9 | 136.2±2.7* | 137.9±3.3* | 139.4±3.8* | 140.4±5.9* | 140.6±7.8* | 139.2±6.9* | 139.4±5.8* |
| **K^+^** | 3.7±0.4 | 4.7±1.1 | 3.7±0.4 | 3.5±0.2 | 3.6±0.6 | 3.5±0.4 | 4.3±1.0 | 4.0±0.4 |
| **Cl^-^** | 112.2±6.9 | 111.0±2.5 | 112.2±4.1 | 112.1±4.7 | 113.8±4.9 | 115.1±7.4 | 124.7±5.2 | 126.9±7.0 |
| **Mg^2+^** | 2.5±0.2 | 3.1±0.7 | 3.0±0.2 | 3.2±0.6 | 3.6±0.4 | 3.5±0.6 | 3.8±0.9 | 3.3±0.9 |
| **Phosphorus** | 5.8±0.9 | 8.3±0.6 | 8.2±2.5 | 7.5±0.9 | 10.7±3.8* | 11.8±5.5* | 11.9±7.4* | 12,4±3.1* |
| **Hematocrit** | 39.5±2.1 | 39.0±1.8 | 38.1±1.5 | 36.2±1.7 | 35.3±2.1* | 35.0±2.3* | 32.9±3.1* | 32.1±2.5* |

ALP – alkaline phosphatase; ALT – alanine transaminase; AST – aspartate transaminase; TB – total bilirubin; DB – direct bilirubin; LDH – high density lipoprotein; TP – total protein; CK – creatine kinase; Ca^2+^ - calcium ion; Na^+^ - sodium ion; K^+^ - potassium ion; Cl^-^ - chloride ion; Mg^2+^ - magnesium ion
